# Mutation enrichment in targeted panels flags immunotherapy-responsive *POLE*-driven hypermutated microsatellite-stable colorectal cancers

**DOI:** 10.1038/s41698-026-01572-1

**Published:** 2026-07-03

**Authors:** Nic Gabriel Reitsam, Kathrin Anna Schneider, Bianca Grosser, Andreas Jung, Daniel Kazdal, Marco Gustav, Jakob Nikolas Kather, Albrecht Stenzinger, Bruno Märkl, Sebastian Dintner

**Affiliations:** 1 Pathology, Faculty of Medicine, University of Augsburg, Augsburg, Germany; 2 Bavarian Cancer Research Center, BZKF, Augsburg, Germany; 3 Else Kroener Fresenius Center for Digital Health, Faculty of Medicine and University Hospital Carl Gustav Carus, TUD Dresden University of Technology, Dresden, Germany; 4 Institute of Pathology, TUM School of Medicine and Health, Technical University of Munich, Munich, Germany; 5 Institute of Pathology, University of Munich, Munich, Germany; 6 German Cancer Consortium (DKTK), partner site Munich, Munich, Germany; 7 Institute of Pathology, University Hospital Heidelberg, Heidelberg, Germany; 8 Department of Medicine I, Faculty of Medicine and University Hospital Carl Gustav Carus, TUD Dresden University of Technology, Dresden, Germany; 9 Medical Oncology, National Center for Tumor Diseases (NCT), University Hospital Heidelberg, Heidelberg, Germany

**Keywords:** Biomarkers, Cancer, Computational biology and bioinformatics, Genetics, Oncology

## Abstract

Pathogenic mutations in the DNA polymerase ε *(POLE)* exonuclease domain define a rare but clinically distinct subset of microsatellite-stable (MSS) colorectal cancers (CRCs) characterized by hypermutation and exceptional immune checkpoint blockade sensitivity. Yet *POLE* testing is not routinely performed, leaving immunotherapy-eligible patients undetected. Because most diagnostic multigene panels do not include *POLE*, strategies enabling its recognition from routine molecular data are needed. We analyzed 675 CRC cases sequenced using a small targeted NGS panel. Tumors with ≥6 non-synonymous SNVs were flagged as potentially hypermutated. Confirmatory *POLE* sequencing and comprehensive genomic profiling (CGP) were performed in preselected cases. Findings were validated using two external *POLE*-mutant CRCs and TCGA-COAD/READ cohorts (>1000 CRCs in total). All *POLE-*mutant CRCs (*n* = 5 of 15 flagged cases; two external validation cases) showed exonuclease domain hotspot mutations, pMMR/MSS status, yet MSI-like histopathology. These tumors exhibited predominantly ultra-high TMB, low dbSNP overlap, C>T transition bias, and disrupted co-mutation patterns - canonical *POLE-*driven hypermutation features. In TCGA, 41/43 *POLE*-mutant CRCs carried panel-detectable co-mutations. Routine small-panel NGS data can flag candidate *POLE*-mutant MSS CRCs for confirmatory testing, enabling detection of immunotherapy-responsive tumors otherwise missed. Integrated with AI-based *POLE*/MSI prediction from H&E slides, this supports multimodal diagnostic workflows enhancing precision immuno-oncology in CRC. Trial registration: NA.

## Introduction

With around 2 million new cases per year, colorectal cancer (CRC) contributes substantially to the global burden of disease^1^—with still limited treatment options and survival chances for those patients, which present with late-stage disease. Nevertheless, personalized and targeted therapies are becoming increasingly available, for which optimal patient stratification and selection is necessary. These treatment options rely on our growing understanding of CRC as clinically and molecularly heterogenous disease, which has enabled the delineation of distinct molecular subtypes^2–6^.

The emergence of immune checkpoint inhibitors (ICI) has significantly improved outcomes in a subset of CRC patients^7–10^. Unfortunately, only the subset of CRCs with microsatellite instability (MSI) or mismatch repair deficiency (dMMR), representing approximately 10–15% of CRCs, are routinely considered to be eligible for immunotherapy^11^. However, there is growing evidence based on molecular studies but also on clinical trial data that some MSS CRCs benefit from ICI therapy as well^8,12^. Identifying these immunotherapy-responsive CRCs is of extraordinary importance.

A small but clinically highly relevant subset of CRCS with DNA polymerase ε *(POLE)* mutations exhibits an ultra-hypermutated phenotype, even in the absence of MSI. This phenotype is mostly driven by functionally significant *POLE* mutations, leading to the accumulation of an extremely high number of mutations (often >100 mutations/Mbp; primarily missense mutations, i.e., single base substitutions that result in the incorporation of a different amino acid or a premature stop codon).

Recently, CRCs with *POLE* mutations have been shown to present with an immunogenic phenotype as well as an exceptional response to ICI-therapy (even better response than dMMR/MSI CRCs)^13–16^, which is particularly relevant given that *POLE*-mutated CRCs almost always have MSS status, and therefore such patients are currently not eligible for immunotherapy and/or are not even tested for *POLE* in routine diagnostics outside of studies anyway, and are thus deprived of a highly effective therapy. As *POLE*-mutant CRCs are relatively rare (approximately 1% of all CRCs)^17^, studying large cohorts is difficult and often not possible.

Interestingly, it has been shown that *POLE*-mutant MSS CRCs show a morphological overlap with MSI CRCs^18^, which has led to the development of an H&E-based dual deep-learning (DL) MSI/*POLE* predictor^19^. Such a test could serve as a prescreening method for further time- and cost-effective genetic testing. Nevertheless, implementing such DL-based pipelines into routine diagnostics has its own challenges, such as coming along with costs for computing power and maintenance as well as relying on a fully digital workflow^20^.

Despite all these findings, routine diagnostic workflows rarely include *POLE* sequencing, particularly in MSS tumors. As a result, these patients may be missed and not considered for potentially beneficial immunotherapy. Moreover, while comprehensive genomic profiling (CGP) can detect *POLE* mutations, it is not available in all settings and may not be indicated for all CRCs. Most centers rely on small, targeted gene panels for molecular profiling, which often exclude *POLE*. Advantages of PCR-based sequencing or targeted sequencing for mutation detection include lower costs as well as faster processing and evaluation.

Ideally, prescreening for *POLE* testing should rely on data already generated during routine diagnostics and therefore not incur additional costs. We believe that for CRCs such an approach is feasible as in most high-income countries the work-up of advanced stage CRCs includes MSI/MMR testing as well as testing for genetic driver mutations, such as *BRAF* or *RAS*^21^, which is nowadays mainly relying on multigene next-generation sequencing (NGS) approaches.

In this study, we investigated whether a high mutational burden observed in small-panel NGS can serve as a pragmatic trigger for *POLE* testing in routine diagnostics. We analyzed a large, single-center CRC cohort, confirmed *POLE* status in selected cases, and validated our approach using external CRC datasets, including The Cancer Genome Atlas (TCGA) cohorts colonic and rectal adenocarcinoma (COAD, READ)^22–24^. We also examined mutational signatures, somatic interactions, and clinicopathological features to better define the diagnostic and therapeutic relevance of *POLE*-driven hypermutation. Our goal was to provide a real-world workflow for identifying immunogenic MSS CRCs that are currently excluded from ICI treatment consideration.

## Results

### Hypermutation in targeted NGS panel approaches as potential hint for underlying POLE-mutations in CRC

In total, 675 CRC cases were analyzed using targeted panel sequencing during the study period in over 84 sequencing runs. To ensure high sequencing quality, we evaluated key quality control (QC) metrics across all cases in the UKA CRC cohort. Most samples had a passing filter read count exceeding the minimum threshold of 200,000 reads, with a few outliers displaying significantly higher or lower read counts. The proportion of Q30 bases, which represents high-confidence sequencing reads, was predominantly above 90%, with only a few cases falling below this threshold. The uniformity of coverage, an important metric for sequencing consistency, showed that most samples maintained a uniformity of ≥90%, while a subset exhibited lower values. The distribution of amplicon mean coverage revealed that most samples had coverage levels well above 1000 reads per amplicon, ensuring sufficient depth for variant detection. Notably, suspected ultra-hypermutated cases, identified by the presence of pathogenic *POLE* mutations, are highlighted in red in the corresponding figures (Supplementary Fig. 1). These cases did not show a distinct deviation from overall QC metrics, suggesting that their hypermutated status was not an artifact of sequencing quality but rather a true biological phenomenon. Cases that did not meet the predefined QC thresholds were excluded (*n* = 71) from further analysis to ensure data reliability and robustness. For further analysis, we focused exclusively on single nucleotide variants (SNVs), excluding insertions and deletions (indels) due to their lower detection reliability in the applied sequencing approach. In the filtered cohort (*n* = 604), we examined the distribution of non-synonymous SNVs (Supplementary Fig. 2). The majority of cases harbored only a few non-synonymous SNVs, whereas a subset exhibited a markedly increased mutational burden (i.e., hypermutated cases). Non-synonymous deletions and insertions were observed at much lower frequencies compared to SNVs, further justifying the focus on SNVs, which are predominantly enriched in established *POLE* mutational signatures^25^. The proportion of SNVs found in the single nucleotide polymorphism database (dbSNP) varied among cases, most of the cases showed a high proportion of SNVs represented in dbSNP, whereas a minor group of cases showed a high proportion of SNVs, which are not documented in the dbSNP database (Supplementary Fig. 2E), indicating a more diverse mutational process with novel and rare variants.

**Fig. 1:**
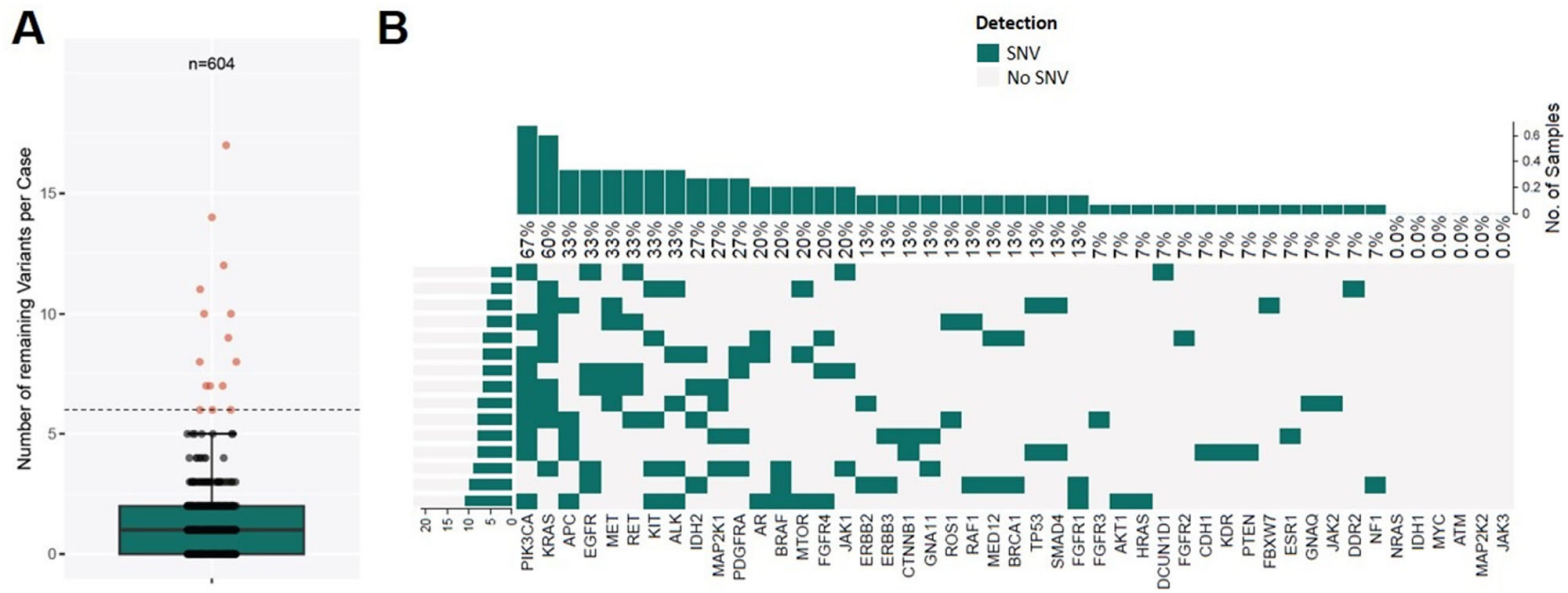
Cases suspected to be ultra-hypermutated due to potential pathogenic *POLE* mutations. In the UKA CRC cohort, cases with an increased number of SNVs (≥6 SNVs) were identified as potential ultra-hypermutated cases. **A** Boxplot displaying the number of remaining SNVs per case. Suspected ultra-hypermutated cases are highlighted in red. The ≥6 SNV threshold is indicated by a horizontal dashed line, highlighting cases with elevated mutation counts. **B** Oncoprint visualization of SNVs in the suspected ultra-hypermutated cases within the CRC cohort. The included SNVs passed quality control and were classified as pathogenic, likely pathogenic, or VUS. The lower panel lists the genes included in the AmpliSeq Focus Panel for Illumina. The left panel displays the number of detected variants per case. The top panel shows the prevalence of variants per gene across the cohort, expressed as a percentage of cases affected. Dark green bars indicate the presence of SNVs in individual cases, while the absence of variants is shown in light gray. CRC colorectal cancer, SNV single nucleotide variant, *POLE* DNA polymerase ε, VUS variant of uncertain significance, UKA University Hospital Augsburg.

Following quality control and the focus on SNVs, we analyzed the genetic landscape of the UKA cohort (*n* = 604) by examining the distribution of pathogenic, likely pathogenic, and variants of unknown significance (VUS) across the targeted genes (Supplementary Fig. 3*, Oncoprint of total UKA CRC cohort*). Of these cases, 162 (27%) showed no detectable alterations, while 442 exhibited characteristic genetic changes. The most frequently altered genes were *KRAS* (44%), *PIK3CA* (20%), *BRAF* (11%), *APC* (9%), and *NRAS* (5%), consistent with known driver mutations in CRC. The mutational patterns across individual cases revealed a heterogeneous landscape, with recurrent alterations in genes involved in key oncogenic pathways such as *MAPK, PI3K,* and *WNT* signaling. The number of detected SNVs per case varied, with a subset exhibiting an increased mutational load, suggestive of hypermutation. The distribution of remaining SNVs per case revealed a distinct bimodal pattern. Most tumors harbored ≤2 pathogenic/likely pathogenic/VUS SNVs, indicating a relatively low mutational burden. However, a subset of cases (*n* = 15, highlighted in red in Fig. 1A) exhibited ≥6 SNVs, significantly exceeding the cohort’s median mutational load.

**Fig. 2:**
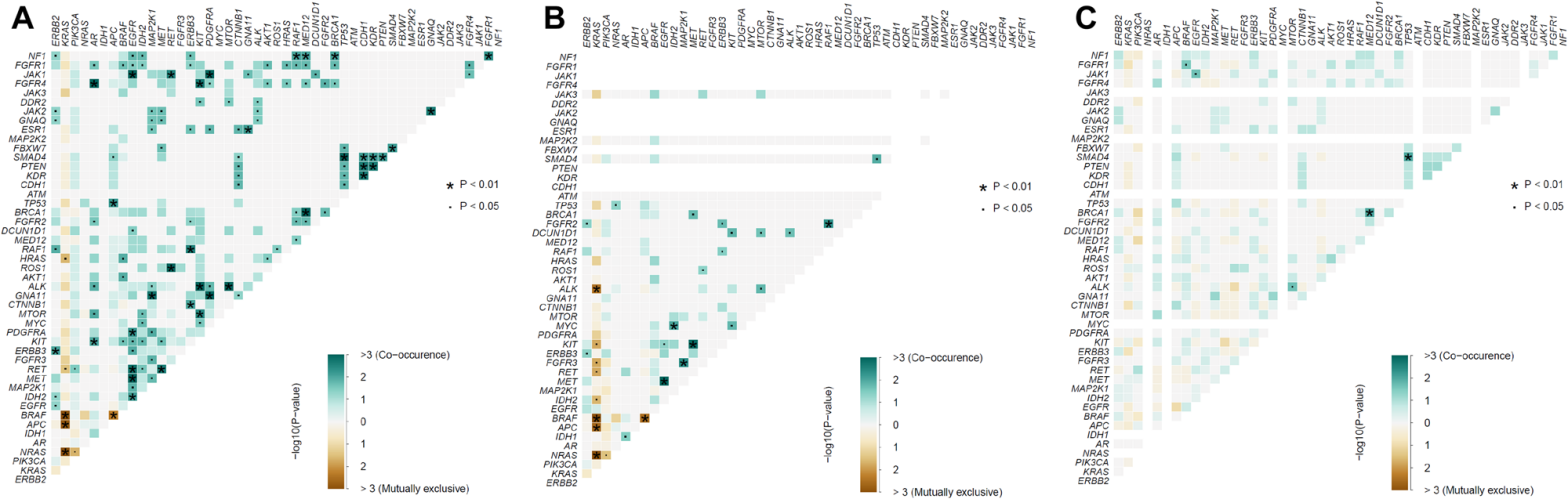
Heatmap representation of somatic interactions between mutated genes in the UKA cohort. The co-occurrence and mutual exclusivity of mutations are visualized with statistical significance indicated by asterisks (*P < 0.01. •*P* < 0.05). Colors represent −log10(*P*-values), with green indicating co-occurrence and brown representing mutually exclusive mutations. Somatic interaction. **A** UKA CRC cohort total. **B** UKA CRC cohort normal. **C** UKA CRC cohort suspected to be ultra-hypermutated. CRC colorectal cancer, UKA University Hospital Augsburg.

In these high-mutation cases, recurrent alterations were observed in *PIK3CA* (67%), *KRAS* (60%), *APC* (33%), and *EGFR* (33%), suggesting a potential distinct oncogenic landscape compared to non-hypermutated cases (Fig. 1B, with higher frequency of *EGFR* alterations, 33% vs 1%). To further characterize the mutational landscape, we analyzed co-occurring and mutually exclusive mutations in the UKA CRC cohort (Fig. 2). In the full cohort, we observed multiple significant co-mutations as well as mutually exclusive alterations, particularly involving *KRAS*. Notably, *KRAS* mutations exhibited strong mutual exclusivity with other key oncogenic drivers such as *BRAF* and *NRAS*, consistent with well-described CRC mutation patterns (Fig. 2A, B). In contrast, the subset of suspected ultra-hypermutated tumors showed fewer significant co-mutations and mutually exclusive relationships. The overall lower number of statistically significant interactions suggests a more diverse, less constrained mutational landscape in these tumors, likely driven by the hypermutation phenotype rather than selective oncogenic pressure but may be also associated to the lower sample size (Fig. 2C).

**Fig. 3:**
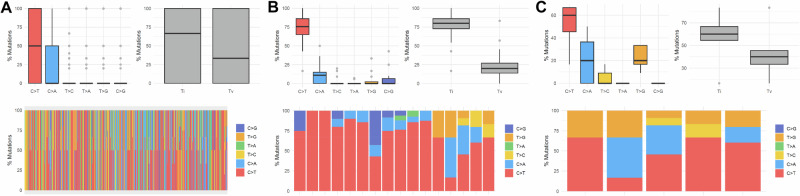
Nucleotide substitution pattern observed in the UKA CRC cohort. **A** UKA CRC cohort normal. **B** UKA CRC cohort suspected to be ultra-hypermutated due to pathogenic *POLE* mutations. **C** UKA CRC cohort ultra-hypermutated. Upper left panel shows nucleotide substitution pattern in general. Upper right panel shows relative distribution of transitions (Ti) and transversions (Tv). Lower panel shows nucleotide substitution pattern per case. CRC colorectal cancer, UKA University Hospital Augsburg, *POLE* DNA polymerase ε.

To further characterize the suspected ultra-hypermutated cases and strengthen the hypothesis of pathogenic *POLE* mutations, we analyzed the nucleotide substitution patterns in the UKA CRC cohort (Fig. 3). In the general CRC cohort, the predominant nucleotide substitution was C>T, followed by C>A, with a relatively balanced distribution of transitions (Ti) and transversions (Tv) with a relevant distribution range (Fig. 3A). In contrast, the subset of (suspected) *ultra-hypermutated tumors* seem to exhibit a marked increase in C>T, C>A and T>G substitutions, with a higher transition-to-transversion (Ti/Tv) ratio (Fig. 3B/C). These findings align with the *known mutational footprint of POLE-driven hypermutation*^26^, reinforcing the hypothesis that suspected ultra-hypermutated tumors in this cohort are likely driven by pathogenic *POLE* mutations.

### Validation of *POLE* mutations in hypermutated cases

**Fig. 4:**
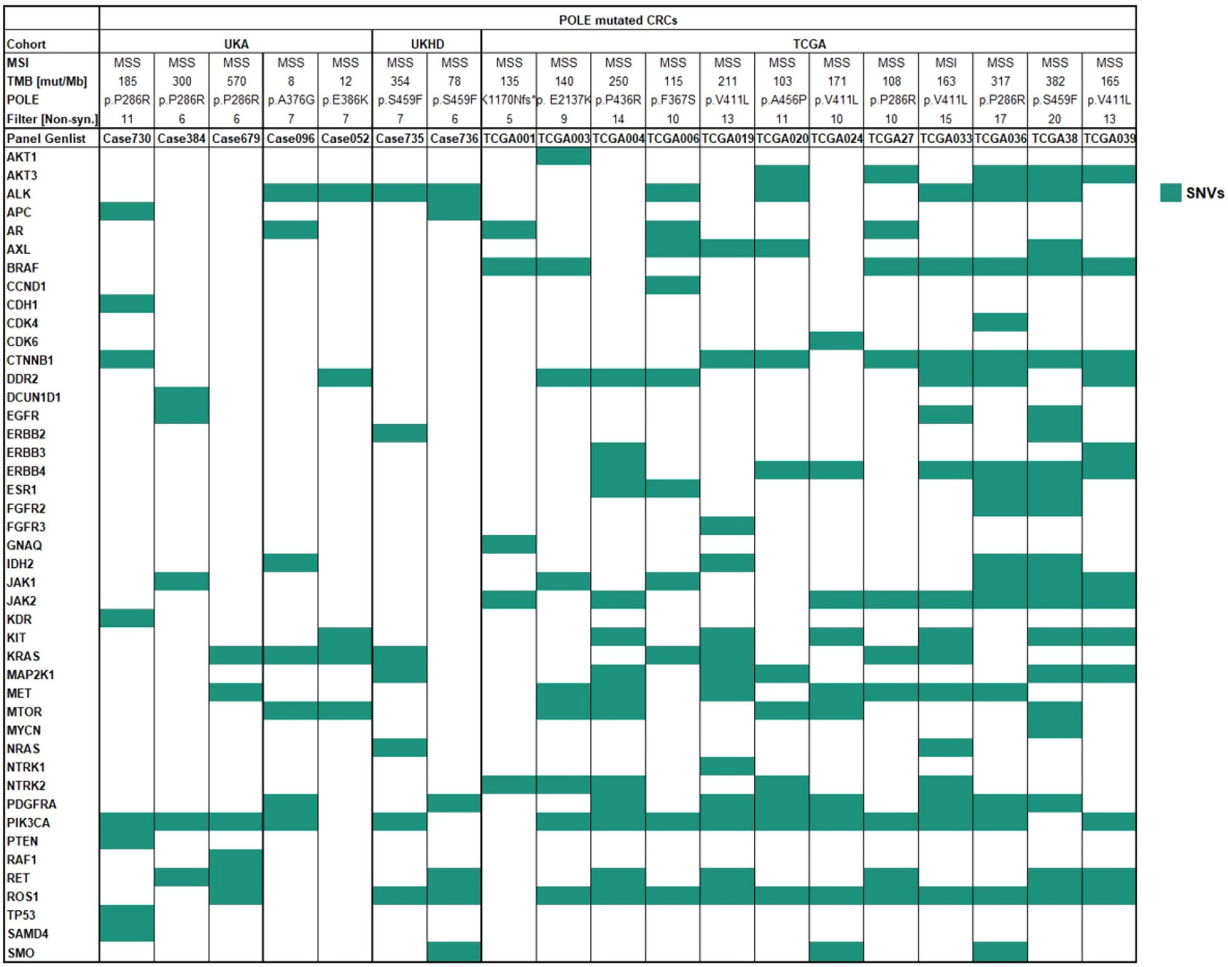
Collection of *POLE*-mutated colorectal cancer cases and mutational landscape of *POLE-*driven ultra-hypermutated tumors. This figure presents a collection of *POLE*-mutated CRC cases from three different cohorts: UKA, UKHD, and TCGA. The UKA cohort includes five cases, characterized by comprehensive genomic profiling (CGP). Additionally, two cases from the UKHD cohort were included for cross-validation. For comparison, ten ultra-hypermutated (TMB > 100 mutations/Mbp) *POLE*-mutated cases from the TCGA-COAD and READ cohort were incorporated. The presence of non-synonymous SNVs across the applied gene panel is highlighted in green, illustrating the distribution of mutations among individual samples. The custom summary data of *POLE*-altered TCGA cases can be found in the *Supplementary Data*. COAD colonic adenocarcinoma, CRC colorectal cancer, *POLE* DNA polymerase ε, UKA University Hospital Augsburg, UKHD University Hospital Heidelberg, TCGA The-Cancer-Genome-Atlas, READ rectal adenocarcinoma.

Given the distinct nucleotide substitution patterns and the elevated transition rate observed in the suspected ultra-hypermutated cases, we hypothesized that these tumors harbor pathogenic *POLE* mutations. To validate this, we performed Sanger sequencing to screen for *POLE* hotspot mutations in the 15 identified outliers, if no CGP was already available as part of the routine work-up of the cases. We proceeded with CGP to further characterize the mutational landscape and assess additional genomic alterations associated with the hypermutated phenotype, like TMB and MSI-score, which mostly show an inverse relationship (Fig. 4).

Three cases exhibited the characteristic p.Pro286Arg *POLE* hotspot mutation in exon 9^9,14^; all these cases were associated with an ultra-hypermutated phenotype (185, 300 and 570 mut/Mbp, with cut-off for ultra-hypermutation >100 mut/Mbp). The other two cases showed a *POLE* exon 12 p.Ala376Gly and p.Glu386Lys, respectively, which were both associated with a borderline hypermutated phenotype (8 and 12 mut/Mbp, with cut-off for hypermutation >10 mut/Mbp). Interestingly, all cases showed at least (≥) 6 variants on a DNA level in a small NGS panel approach (Supplementary Table S1), indicating here a positive correlation between panel SNV count and TMB. Analysis of non-synonymous SNVs using the targeted gene panel revealed recurrent mutations in *PIK3CA*, *KRAS*, *APC*, *TP53*, and *FGFR*, with alterations frequently occurring in genes involved in the MAPK and PI3K signaling pathways. This mutation pattern is also reflected in the somatic interaction analysis (Fig. 2C), which revealed a notably reduced number of significant co-mutations and mutually exclusive alterations in the suspected cases compared to the overall cohort. *KRAS* mutations, which typically exhibit strong mutual exclusivity with other driver mutations, showed a loss of this exclusivity in *POLE*-mutated tumors (Fig. 4). These findings support that the suspected ultra-hypermutated cases in the UKA cohort exhibit a distinct genomic profile, characterized by high TMB, MSS status, and the disruption of typical co-mutation and mutual exclusivity patterns, consistent with *POLE*-driven hypermutation. We therefore hypothesize that such an enrichment of molecular alterations on a DNA level in pMMR/MSS may serve as a practical trigger for confirmatory *POLE* testing, as pathogenic *POLE* mutations were identified in approximately one third of suspected cases (5/15). Sensitivity and specificity cannot be estimated for the full cohort because *POLE* status was not uniformly assessed in screen-negative tumors.

To support our hypothesis, we cross-validated with two additional *POLE*-mutated CRC cases from Heidelberg University (UKHD), both carrying the *POLE* p.S459F variant (Fig. 4). Like the UKA *POLE*-mutated cases, these tumors exhibited a MSS phenotype and a corresponding high TMB (354 and 78 Mut/Mbp), reinforcing their hypermutated status. Immunhistochemical pMMR status with retained expression of MLH1, PMS2, MSH2, and MSH6 was confirmed (Supplementary Fig. 4). The mutational landscape of these cases, assessed using the targeted gene panel, revealed alterations in *APC*, *PIK3CA*, and *TP53*, aligning with the patterns observed in the UKA cohort (Fig. 4).

### Validation in TCGA cohort

To further verify that *POLE*-mutant CRCs show, due to their biology, a substantial number of DNA alterations even when tested with a focused NGS panel, we studied the publicly available TCGA-cohorts COAD and READ for all cases with any *POLE* variant (*n* = 43, 7% prevalence of any POLE variant; nota bene: the functional relevance of non-exonuclease domain *POLE* variants is still a matter of ongoing debate), and investigated if these cases also show concurrent co-mutations in genes that are covered by the AmpliSeq for Illumina Focus Panel, which is a small NGS panel that is routinely used for genetic testing of driver mutations in CRC cases. Here, all but two cases showed several potentially detectable co-mutations (Supplementary Figs. 5 and 6). The two cases with no potentially detectable co-mutation showed a low TMB, and conventional adenocarcinoma NOS morphology *(TCGA-AA-3678, POLE* p.D1752N*)* with in one case partial mucinous differentiation (*TCGA-NH-A50U, POLE* p.A2040V; see Supplementary Fig. 7).

The top co-mutated genes in *POLE*-altered TCGA-CRC cases detectable by the AmpliSeq for Illumina Focus Panel were *ROS1* (51%), *PIK3CA* (49%), *BRAF* (39%) and *ERBB4* (37%); we also observed alterations in these genes in our own *POLE*-mutated cases.

We again analyzed the transition-to-transversions (Ti/Tv) ratio in those 41 CRCs in TCGA with *POLE* variants and concurrent mutations detectable by a small panel (Supplementary Fig. 8). Those CRCs with *POLE* variants predominantly displayed C>T transitions here, and which was also true for our own UKA cohort *(*Fig. 3*)* and has already been closely linked to defective proofreading in *POLE*-mutant cancers^26^. Additionally, by analyzing somatic interactions within TCGA CRC cases with *POLE* variants and concurrent, potentially detectable mutations, we found, e.g., significant *JAK2-ERBB4*, *PDGFRA-ERBB2*, *ETV1-JAK*, *ROS1-ETV1*, and *PDGFRA-JAK2* co-mutations (all adjusted *p*-values < 0.001; Supplementary Fig. 9).

**Fig. 5:**
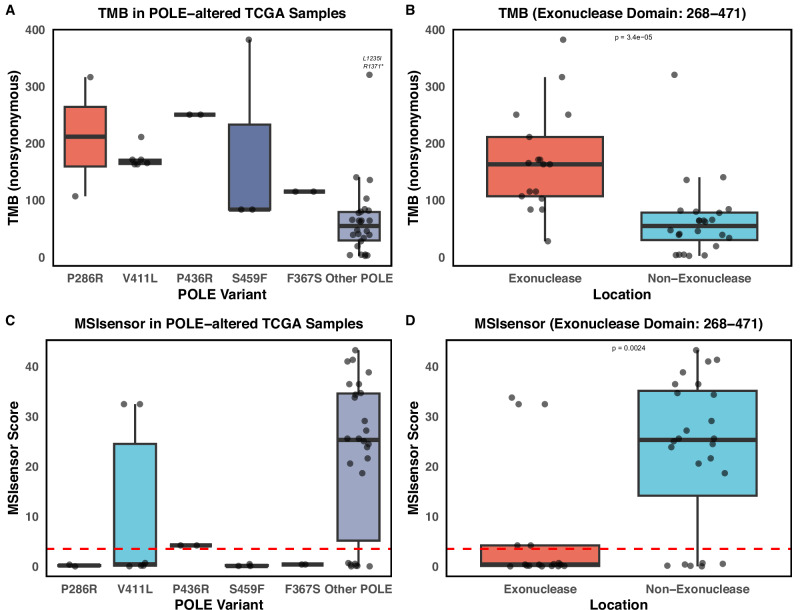
TMB and MSIsensor scores of POLE-altered TCGA-CRCs stratified by *POLE*-variant or location of *POLE* alteration (exonuclease domain vs non-exonuclease domain). **A** Known *POLE* hotspot mutations are associated with high TMB (i.e., hypermutated phenotype). We observed P286R and S459F variants in our *POLE*-mutated CRCs associated with hypermutation. **B** *POLE* alterations located in the exonuclease domain (amino acids 268–471) are linked to higher TMB compared to non-exonuclease domain alterations (*p* = 3.4e-05, Wilcoxon test). **C** Most other *POLE*-alterations occur in the presence of MSI. Hotspot mutations are rarely linked to high MSIsensor scores (*POLE* V111L). **D** CRCs with *POLE* alterations in the exonuclease domain display significantly lower MSIsensor scores (*p* = 0.0024, Wilcoxon test). Red dashed line in (**C**, **D**) highlights a MSIsensor score of 3.5, which can be considered as cut-off between MSI and MSS^30,31^. Samples with at least one mutation in the exonuclease domain were considered as Location = Exonuclease. CRC colorectal cancer, MSI microsatellite instability, *POLE* DNA polymerase ε, TMB tumor mutational burden, TCGA The-Cancer-Genome-Atlas.

Consistent with our findings, we observed a strong association between known *POLE* hotspot mutations and hypermutation in TCGA-CRC (any *POLE* variant, *n* = 43), with alterations in the exonuclease domain (amino acids 268–471)^27^ linked to significantly higher TMB (*p* = 3.4e-05, Wilcoxon test) and lower MSIsensor scores (*p* = 0.0022, Wilcoxon test) compared to non-exonuclease domain alterations (Fig. 5). Notably, most other *POLE* alterations co-occurred with MSI, whereas hotspot mutations were rarely linked to high MSIsensor scores. Nevertheless, some *POLE* variants outside of the exonuclease domain were associated with high TMB scores, such as p.L1235I/R1371* or p.E2137K. For a detailed list of non-exonuclease *POLE* alterations and corresponding TMB scores refer to Supplementary Table S2.

To assess whether mutation enrichment within the 52-gene panel reflects genome-wide mutational burden, we performed an in-silico validation using TCGA-COAD/READ somatic mutation data (Supplementary Fig. 10). Across all CRCs, the number of panel-detectable SNVs correlated strongly with exome-wide TMB (Spearman *ρ* = 0.673, *p* < 0.0001). Notably, within *POLE*-altered tumors, panel SNV counts showed an even stronger association with TMB (*ρ* = 0.904, *p* < 0.0001), indicating that panel mutation enrichment closely reflects the degree of polymerase-driven hypermutation. Consistent with this observation, panel SNV burden increased stepwise across TMB categories (<10, 10–99, ≥100 mut/Mbp; Kruskal-Wallis *p* < 0.0001).

Characteristics of all TCGA-CRCs with *POLE* alterations (*n* = 43) and a detailed list of co-mutations in genes detectable with the AmpliSeq for Illumina Focus Panel are available in the Supplementary Data 1, which provides interesting insights how many co-mutations maybe detectable with a small NGS approach.

### *POLE*-mutant CRCs: characteristic clinical features and MSI-like morphology

**Fig. 6:**
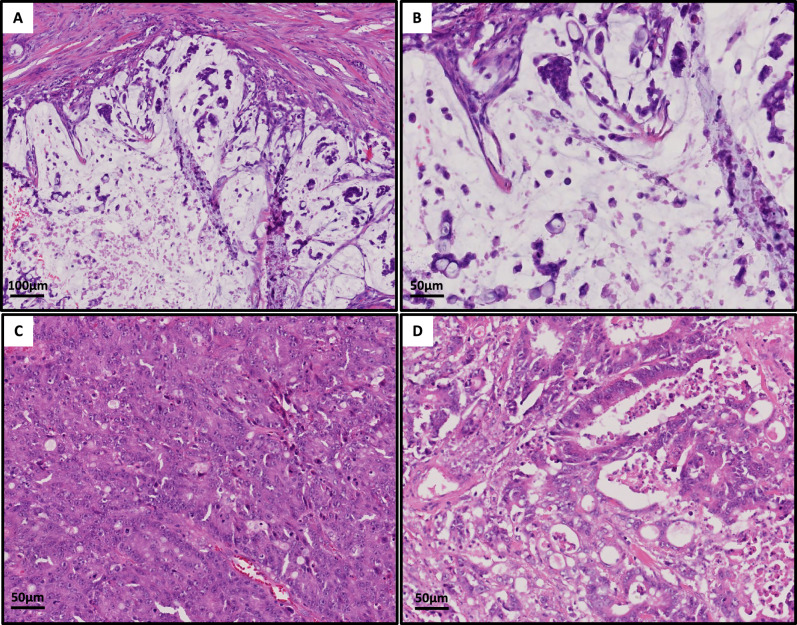
Histomorphological spectrum of *POLE-*mutant CRCs. The *POLE*-mutant CRC cases of our cohort exhibited distinct morphologic features, which have been previously linked to POLE-mutant CRCs and show an overlap with a “MSI-like” morphology, like mucinous histology (**A**, **B**), signet ring cells (**B**) or medullary morphology (**C**). However, in some cases these specific differentiation was only a partial component of a conventional adenocarcinoma NOS (**C**, **D**). CRC colorectal cancer, MSI microsatellite instability, NOS not otherwise specified, *POLE* DNA polymerase ε.

It is already known that *POLE* mutations are quite rare in CRC, which was also the case in our academic centers. Interestingly, most of those cases showed at least a partial mucinous/signet ring cell or medullary morphology (Fig. 6). Moreover, all our patients with *POLE*-mutant CRCs were male, and the tumors were often right-sided.

A summary of our seven *POLE*-mutant CRCs is provided in Supplementary Table S1.

### Potential workflow to identify *POLE*-mutant CRCs in routine practice

**Fig. 7:**
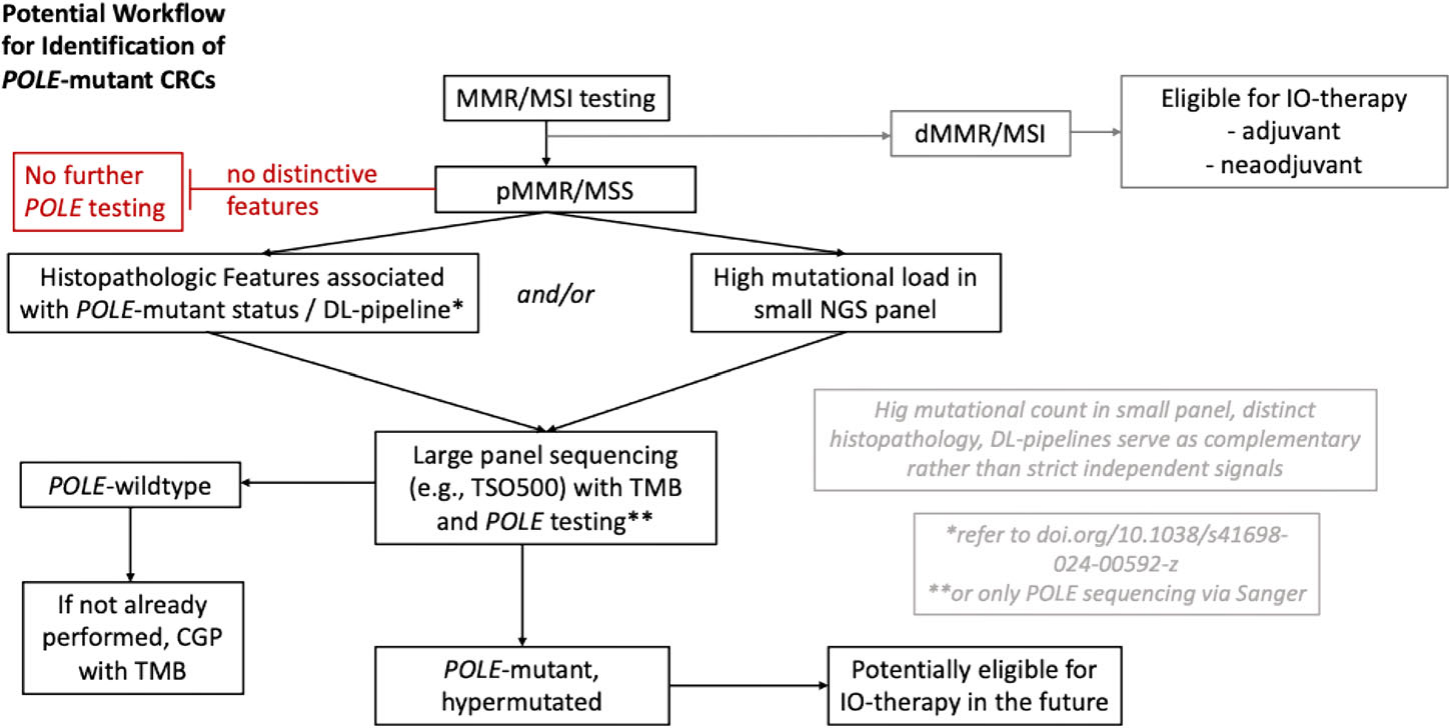
Potential workflow to identify *POLE*-mutant CRCs in routine pathology practice. MMR-protein or PCR-based MSI testing is already widely implemented in the routine workup of CRC cases. If a case is dMMR/MSI, IO therapy approaches are already approved (depending on the disease stage) and will be considered. If a case is pMMR/MSS but shows histopathologic features linked to MSI/*POLE*-status, like medullary or mucinous differentiation, *POLE* testing should be considered—especially if the patient shows certain characteristics that are associated with *POLE*-mutations in CRC, such as right-sided tumors, young age and/or male gender. Moreover, we previously have already published a dual *MSI/POLE* DL-based pipeline relying solely on H&E-morphology. If a CRC case undergoes NGS via a small panel, which is commonly used in routine practice (like Oncomine Precision [ThermoFisher] or Hotspot [Illumina]), and the case exhibits an unusually high number of detected variants, this is also suspicious for a *POLE* alteration. In all cases, *POLE* testing should then be performed, either via Sanger sequencing or via a broad NGS-based panel approach (like TSO500), which would also provide additional interesting information, e.g., TMB. If the CRC shows a (pathogenic) *POLE*-mutation and/or hypermutation, this patient should be considered for IO-therapy—despite pMMR/MSS status. The proposed criteria (mutational load, specific histology, DL-based pipeline) should be interpreted as complementary signals rather than strict independent triggers. CRC colorectal cancer, DL deep learning, IO immune oncology, MSI microsatellite unstable, MSS microsatellite stable, (p)MMR (proficient) mismatch repair, *POLE* DNA polymerase ε, TMB tumor mutational burden.

Based on our current and previous findings, in which we could show that a dual DL-based *POLE*/MSI prediction on routine H&E-stains is feasible, we propose here a workflow to better identify this important CRC subtype (Fig. 7).

We propose that all pMMR/MSS CRCs with a typical MSI-like/*POLE* morphology, as seen in our cases (Fig. 6) should be subjected to *POLE* testing. Ideally, these cases also have the typical clinicopathological characteristics that we (Supplementary Table S1) and others (in particular *Domingo* et al*.*) have described: these tumors often manifest in young male patients with right-sided location^13^. With access to a DL-based *POLE*/MSI predictor, as for example published by us^19^ or to DL-based MSI predictors already commercially available (such as *MSIntuit* from Owkin^28^), which are becoming increasingly prevalent, these cases are likely to be conspicuous here, namely as MSI/*POLE* predicted despite immunohistochemical or PCR-based pMMR/MSS status. In the current work, we established an enriched number of detectable alterations in small NGS-based panels that do not cover *POLE* (Fig. 4) as suggestive of *POLE* alterations in pMMR/MSS CRC. All these indicators may justify targeted *POLE* testing so that patients can then undergo effective immunotherapy if necessary.

## Discussion

Immunotherapeutic approaches have fundamentally changed the way certain solid cancer entities are treated and have tremendously improved patient outcomes^7,29^. Unfortunately, in CRC only the small subset of dMMR/MSI CRCs, which are less than 10% of all metastatic CRCs^11^, are eligible for ICI-therapy. Strikingly, a rare subgroup of pMMR/MSS CRCs is characterized by mutations in the *POLE* gene (around 1% of all CRCs^13,30^), which is characterized by hypermutation, high immunogenicity and hence an excellent responsiveness to IO-therapies^13–15,17,31,32^—even higher than in dMMR/MSS cases^15^. Since most *POLE*-mutant CRCs are pMMR/MSS and testing for *POLE* mutations is not routine in CRC, these patients are missing a critical treatment opportunity.

Just recently, *Ambrosini* et al*.* could prove in a cohort of 27 patients with metastatic CRCs (mCRCs) with *POLE/D1* proofreading deficiency that these patients exhibit even more favorable outcomes when treated with ICI compared to dMMR/MSI-H mCRC patients^15^, which also show good therapy responses upon ICI treatment^7^.

In Europe and the US, small targeted NGS panels are currently routinely used for CRC cases in which molecular profiling is clinically indicated, i.e., in locally advanced, metastatic or recurrent settings. Panel-based testing is currently not intended as universal screening for every early-stage CRC, but as part of routine molecular work-up when predictive biomarkers are required^21^.

In our local cohort, we identified 15 CRCs exhibiting a notable enrichment of molecular alterations within a small gene panel. Subsequent testing revealed that five of these cases harbored *POLE* mutations. This observation suggests that the well-established high mutational load characteristic of *POLE*-mutated CRCs can be effectively detected using clinically employed small panel approaches. Even though we did not perform CGP for the further nine suspicious cases, these might be hypermutated due to other alterations in the DNA repair, e.g., *POLD1* mutations. To substantiate this hypothesis, we analyzed two additional external *POLE*-mutant CRC cases from Heidelberg University, both carrying the *POLE* p.S459F variant. Consistent with our initial findings, these cases demonstrated a similar enrichment of detectable alterations when assessed with a NGS panel. Furthermore, our examination of TCGA cohorts, specifically the COAD and READ datasets^22–24^, revealed that most CRCs with detectable *POLE* variants possessed co-mutations identifiable by conventional small routine NGS panels. Interestingly, the pronounced co-mutation patterns observed in the overall cohort were largely absent in the suspected hypermutated cases. This finding indicates that hypermutation may disrupt typical oncogenic dependencies. Notably, the mutual exclusivity commonly observed in *KRAS* mutations with other driver mutations was significantly reduced in the suspected hypermutated tumors. This observation aligns with previous studies reporting a high prevalence of atypical *KRAS*, *NRAS*, and *BRAF* mutations in *POLE*-hypermutated CRCs, suggesting that the hypermutated environment fosters a unique mutational landscape^32–34^.

Three of our five cases in the exploration cohort (60%) exhibited a characteristic *POLE* p.Pro286Arg hotspot mutation^14,35^. Nevertheless, understanding the exact biologic and predictive role of *POLE* variants is still an evolving topic^31,36^ as only a few hotspot variants in or close to the exonuclease domain of *POLE/POLD1* should be considered as oncogenic (the exonuclease domain of *POLE* is located between amino acid residues 268 and 471^27^), leading to DNA repair defects and a high TMB^37^. However, in TCGA-CRC there are some samples with non-exonuclease domain *POLE* mutations and high TMB values (summarized in Supplementary Table S2), highlighting the need for further studies investigating the association between *POLE* variant, hypermutation, immunogenicity and therapy response.

Importantly, the number of non-synonymous SNVs detected by the 52-gene panel correlated with the hypermutated phenotype assessed by CGP-derived TMB in cases with available CGP and in the TCGA cohort. Nevertheless, we do not propose small-panel mutation counts as a replacement for formal TMB measurement, but as an easily accessible routine diagnostic trigger to select MSS CRCs for confirmatory *POLE/POLD1* testing and CGP.

Additionally, our cases together with the literature provide the basis for a morphologically and clinically guided genotyping. Young patients with non-MSI CRCs should be tested for *POLE* mutations as sporadic but also germline *POLE* variants are known to contribute to a relevant subset of early-onset CRC^13–15,30^. Moreover, right-sided tumor location as well as male gender can be regarded as clinical indicators of underlying *POLE*-mutations in pMMR/MSS CRCs, especially if they occur in combination with other hints. It has already been described by *Shia* et al*.* for the TCGA COAD and READ cohorts that *POLE*-mutant CRCs display morphologic similarities with MSI CRCs, also displaying often mucinous or medullary morphology^18^. The fact that morphological features can help to draw conclusions about *POLE* status has also been shown for endometrial carcinoma (EC). In EC, it was shown that the morphological appearance of morules suggests a *POLE* wild-type status^38^. Additionally, *POLE*-mutant ECs are linked to higher-grade EC, abundant TILs and presence of giant cells, which shows that morphologically guided genotyping to identify *POLE*-mutant cancers may not restricted to CRC^39–41^.

The histologic similarity between MSI and *POLE*-mutant CRCs motivated a previous study where we could show that a DL-based model initially trained to identify MSI CRCs shows also good performance in flagging *POLE*-mutant CRCs^19^. Beyond providing a paradigmatic example that morphologic overlaps could be helpful to predict rare genotypes via DL-based algorithms, this could be another potential use case for DL-based MSI classifiers^28,42–44^, which may be soon implemented into routine diagnostic pathology^20,45,46^. Our previously published dual DL-based *POLE*/MSI predictor seemed to achieve even better performance when focusing on *POLE* p.P286R mutations^19^.

Taking all our current and previous findings into account, we tried to establish a potential multimodal workflow (Fig. 7) to increase the detection rate of *POLE*-positive CRCs in a routine setting. Each of the mentioned indicators, namely a high mutational load in a small NGS panel approach, specific morphologic and/or clinicopathological features and/or MSI/*POLE* prediction via DL-based algorithms based on H&E WSI should trigger *POLE* testing to prevent these patients from missing out on highly effective therapies.

We recognize that large panel or even exome testing is not possible/available in many situations, and especially not in all areas globally. There are still large disparities in the deployment of genomic testing^47^. For DL-based MSI/*POLE* predictors the same is true. Nevertheless, as these DL models are based on digitized H&E WSI, in principle such solutions would be cost-effective and could be made available to low-resource healthcare settings via remote solutions.

Even in highly developed health care systems, the cost-effectiveness of large panel, whole exome or even whole genome testing remains unclear and certainly context dependent^48^.

Even though our study is based on a large internal cohort as well as the publicly available TCGA dataset and provides a pragmatic diagnostic workflow, several limitations must be acknowledged: Our proposed threshold of ≥6 SNVs detected in a small panel is exploratory in nature and has not been statistically confirmed or optimized. Additional, independent studies should determine the optimal cut-off. The exact numerical threshold may vary across institutions depending on panel composition, sequencing platforms, and bioinformatic variant calling and interpretation workflows, although the general concept of increased mutational burden within a small, targeted panel as an indicator of *POLE*-associated hypermutation is likely to remain applicable. The SNV counts underlying the ≥6 threshold include VUS alongside pathogenic and likely pathogenic variants, which is biologically justified given that mutational accumulation itself constitutes the signal in *POLE*-driven hypermutation, but introduces variability inherent to VUS classification across laboratories and annotation databases.

Moreover, the number of confirmed *POLE*-mutant cases in our cohort is inherently limited, reflecting the well-established rarity of this tumor subtype in CRC. This restricts the statistical power of comparative analyses and limits the generalizability of our findings. Accordingly, all conclusions should be interpreted as exploratory and hypothesis-generating, and the proposed workflow requires prospective validation in larger, ideally multicentric cohorts. Furthermore, since *POLE* testing was not systematically performed in screen-negative cases, sensitivity and specificity of the proposed screening approach cannot be formally calculated, and the false-negative rate remains unknown. No clinical outcome data, such as response to immune checkpoint inhibition or survival, were available for the confirmed *POLE*-mutant cases, which precludes any assessment of the workflow’s direct therapeutic impact. Sequencing platform and downstream analysis heterogeneity across the internal, external, and TCGA cohorts may introduce technical variability that limits direct comparability of mutation counts. Finally, potential FFPE-associated C>T deamination artifacts cannot be entirely excluded, although stringent quality filtering and the markedly elevated mutation burden in affected cases argue against a purely artifact-driven explanation.

While *POLE* exonuclease domain mutations and their association with an ultra-hypermutated phenotype have been previously described, most existing data originate from large-scale genomic studies or clinical trials using CGP. In contrast, our study focuses on a real-world diagnostic setting where small, targeted NGS panels are routinely used (often without *POLE* coverage). This creates a diagnostic blind spot for identifying MSS CRCs with high immunogenic potential. In the long term, routine CRC panels may benefit from incorporating the exonuclease domains of *POLE* and *POLD1*, as pathogenic alterations in these genes, together with TMB, have emerging relevance for immunotherapy selection^15^. However, many diagnostic laboratories currently rely on established targeted panels that do not include these loci. While CGP is increasingly adopted, its routine use as reflex testing for all CRC samples remains limited in many settings due to cost, infrastructure, and workflow considerations within already resource-constrained healthcare systems. Hence, our proposed workflow will continue to be relevant, particularly in decentralized diagnostic environments where broad CGP is not universally available.

Furthermore, we propose an integrative workflow that combines SNV burden, MSS status, histologic features, and mutational signature analysis, elements that are readily accessible in clinical pathology, to flag cases for further *POLE* testing. To our knowledge, this is the first study to evaluate and validate this approach across internal, external, and public datasets. While large databases, such as TCGA, have provided comprehensive molecular insights into CRC subtypes^24^, our study integrates our institutional CRC cohort with TCGA data to derive clinically applicable diagnostic guidance that may facilitate the identification of immunogenic *POLE*-mutant CRCs in routine practice.

## Methods

### Ethical approval and study cohort

The study was approved by the ethical committee of Ludwig Maximilian University (LMU) of Munich (reference: project number **24-0780**, short title **InMutRat**) and was performed in accordance with the Declaration of Helsinki. Informed consent was waived due to the retrospective nature.

Our total internal cohort (UKA cohort) comprised 675 CRCs that underwent targeted panel sequencing at the University Hospital Augsburg (Institute for Pathology and Molecular Diagnostics). The *POLE*-mutant cohort consisted in total of seven CRC cases from three different academic medical centers (University Hospital Augsburg [UKA], LMU Munich via round robin quality control test, University Hospital Heidelberg). Different material was used for downstream analysis (primary resection specimens, superficial biopsies as well as biopsies of liver metastasis).

### Histopathology and immunohistochemistry

Surgical CRC specimens were promptly fixed in 4% buffered formalin for a minimum of 12 h. After routine processing, 2 µm sections were cut from each specimen and stained with H&E (Merck, Darmstadt, Germany). Additionally, immunohistochemical (IHC) stains were also performed on 2 µm whole slide sections using primary antibodies for MLH1 (Clone: M1, Roche [Basel, Switzerland], RTU), PMS2 (Clone: EP51, Agilent Technologies [Santa Clara, CA, USA], RTU), MSH2 (Clone: G219-1129, Roche [Basel, Switzerland], RTU) and MSH6 (Clone: EP49, Leica Biosystems [Newcastle, UK], RTU) in a fully automated manner on a Ventana BenchMark ULTRA platform with an iVIEW DAB detection system (Roche, Mannheim, Germany). Loss of MMR protein expression was defined as complete absence of nuclear staining in tumor cells for at least one MMR protein (MLH1, PMS2, MSH2, or MSH6), with intact staining in internal control cells. For further information, please also refer to our previous publication^49^.

### Molecular pathology

#### Sanger sequencing

For targeted *POLE* analysis, Sanger sequencing reactions were performed using the BigDye™ Terminator v3.1 Cycle Sequencing Kit (Applied Biosystems) and analyzed on an automated capillary sequencer (SeqStudio Genetic Analyzer, Thermo Fisher). The resulting sequences were evaluated with Geneious® software (Geneious® 10.0.9, Biomatters Ltd., https://www.geneious.com) and aligned to the *POLE* reference sequence (RefSeq: NM_006231). Variants were classified based on the guidelines provided by the American College of Medical Genetics and Genomics (ACMG)^50^. The *POLE* (OMIM 174762) exon 9, 11, 13, and 14 region was amplified by polymerase chain reaction (PCR) using specific primers. PCR reactions were carried out in a total volume of 15 µl.

#### AmpliSeq for illumina FOCUS panel

Tumor DNA was extracted from formalin-fixed, paraffin-embedded (FFPE) tissue samples using Maxwell® FFPE Plus DNA Kit (Promega, Madison, Wisconsin, USA) following the manufacturer’s instructions. The concentration and quality of the extracted DNA were assessed using a Quantus (Promega, Madison, Wisconsin, USA) fluorometer.

For this study the multiplex PCR based AmpliSeq for Illumina Focus Panel was used (Illumina, San Diego, California, USA). The panel consists of 269 primer pairs for the detection of hot-spot mutations in 52 cancer-related genes on DNA. Amplicon library preparation was performed using approximately 10 ng of DNA, as recommended by the manufacturer. The final library was purified using AMPure XP magnetic beads (BeckmanCoulter, Krefeld, Germany) and was quantified using QuantiFluor® ONE dsDNA System (Promega, Madison, Wisconsin, USA). The individual libraries were diluted to a final concentration of 9 pmol/L, pooled and processed for sequencing by synthesis using a MiSeq reagent kit V2 (300 cycles) on a MiSeq System (Illumina, San Diego, California, USA).

Secondary analysis was performed using the application Generate FASTQ (Version 2.0.01.17; RUO) and DNA Amplicon (Version 2.1.0.19; RUO) on the Local Run Manager (Version 1.0.0.7; Illumina, San Diego, California, USA). The reads were aligned to the human reference sequence build hg19. Detection of SNVs and indel polymorphisms, relative to the human reference sequence, was performed using the BaseSpace Variant Interpreter (Illumina, San Diego, California, USA).

#### TSO500

For verification of *POLE* mutation and correlation with TMB status, we used the TruSight Oncology 500 (TSO500) panel (Illumina). Library preparation was performed using the hybrid capture-based TruSight Oncology 500 Library Preparation Kit (Illumina) following the manufacturer’s protocol. Finally, the libraries were pooled, denatured, and diluted to the appropriate loading concentration. TSO500 libraries were sequenced on a NextSeq™ 550Dx system (Illumina). For secondary analysis raw data were analyzed using DRAGEN TruSight Oncology 500 Analysis Software 2.5.2 on a local DRAGEN server (Illumina).

Sequence variants were described using HGVS nomenclature^51^. Variant classification was based on the guideline of the American College of Medical Genetics and Genomics (ACMG)^50^. Hypermutation was defined as >10 mut/Mbp and ultrahypermutation as >100 mut/Mbp, based on established literature^52^.

### Data processing and variant classification

To analyze variant data from CRC patients in the UKA cohort, we developed an R-based pipeline (version 4.3.0) with filtering criteria based on sequencing quality, variant-specific parameters, and variant interpretation.

Quality Filtering: Samples were included if the mean amplicon coverage was ≥1000, base coverage uniformity ≥90%, and Q30 bases ≥90%. Variants were excluded if they failed quality thresholds (genotype quality = 100, total read depth ≥200, alternative allele depth ≥10, QC-filter = “PASS,” VAF > 0.03) or had transcript consequences classified as “synonymous,” “intron,” “upstream,” “downstream,” or “3’ UTR” variants.

Variant Interpretation: A hierarchical prioritization approach was applied. First, ClinVar (version 20180129) annotations were assessed. Variants classified as “benign” or “likely benign” were excluded, while “pathogenic,” “likely pathogenic,” and “uncertain significance” variants were retained. If no ClinVar entry was available, population databases—gnomAD (version 2.0.2), gnomAD Exome (version 2.0.2), and TOPMed (version freeze_5)—were queried, and identified variants were excluded. If absent in these databases, in silico tools PolyPhen and SIFT were used, excluding variants predicted as “benign/tolerated” or “NA” and retaining those classified as “probably/possibly damaging” (PolyPhen) or “deleterious” (SIFT). The analysis was performed using the R packages “tidyverse,” “jsonlite,” “readxl,” “writexl,” “tools,” “future,” “future.apply,” and “magrittr.” The most frequently altered genes in the cohort were visualized with the oncoPrint function from the “ComplexHeatmap” R package. To analyze mutation co-occurrence, a custom function was developed, like the *somaticInteractions* function in the “maftools” package (https://rdrr.io/bioc/maftools/man/somaticInteractions.html). The *titv* function (“maftools” package) was employed to calculate and visualize the transition/transversion (TiTv) ratio in the dataset. All analyses and visualizations were conducted using the R packages “ggplot2,” “ComplexHeatmap,” and “maftools” using R version 4.4.0.

### *POLE* in TCGA-COAD & READ

Somatic mutation data from The-Cancer-Genome-Atlas (TCGA) cohorts for colon and rectum adenocarcinomas (COAD and READ)^24^ were accessed through *TCGAbiolinks* and downloaded using the *GDCquery* and *GDCdownload* functions. The mutation data for these genes were accessed and the *oncoplot* function was used to visualize the most frequent mutations in these genes among *POLE*-altered samples. We specifically focused on the genes (*n* = 52) that are covered by the AmpliSeq for Illumina Focus Panel as an example for a small targeted NGS panel approach used in CRC and hence used the TCGA samples for a “virtual” small panel simulation. In our own UKA CRC cohort occasionally variants in other genes were called (“off-target calls”), and hence we also looked at the union between the genes and the gene list and the additional genes (*n* = 64). The *somaticInteractions* function from “maftools” was applied to assess gene interaction patterns and mutation co-occurrence. The *titv* function was used to calculate and visualize the transition/transversion (TiTv) ratio in the data. Sample IDs for *POLE*-mutated samples were exported for further analysis.

A MSIsensor score of 3.5 can be considered as cut-off between MSI and MSS^53,54^.

Further information on the *POLE*-altered CRCs in TCGA can be found in Supplementary Table 2 as well as the *Supplementary Material*.

R packages used were analysis of TCGA data were “TCGAbiolinks,” “maftools,” “dplyr,” “tidyr,” “ggsci,” “FSA” and “ggpubr”.

For further information and available code refer to https://github.com/ngr-path/TCGA_POLE_CRC_Concurrent or contact the corresponding authors (NGR, SD).

### Statistical methods

Relative frequencies are given as percentage (%). Group comparisons of continuous variables were performed using the Wilcoxon rank-sum test. A *p*-value < 0.05 was considered statistically significant. Mutation co-occurrence was assessed using the *somatic Interactions* function from the “maftools” package, which applies Fisher’s exact test and corrects for multiple testing internally (Bonferroni correction). For further details on statistical analyses and data processing, please refer to the relevant sections above. All statistical analyses were conducted using R (version 4.4.0).

## Supplementary information

Supplementary information

Supplementary data 1

## Data Availability

For further information and available code refer to https://github.com/ngr-path/TCGA_POLE_CRC_Concurrent or contact the corresponding authors (NGR, SD). TCGA-COAD and READ cohorts can be accessed via *cBioPortal*. Due to privacy reasons, the UKA cohort is not publicly available.
