# Supplementary material for: Mutation enrichment in targeted panels flags immunotherapy-responsive POLE-driven hypermutated microsatellite-stable colorectal cancers
Source: NPJ Precis Oncol. 2026 Jul 3;10:263. doi: 10.1038/s41698-026-01572-1 (PMC13346926; doi:10.1038/s41698-026-01572-1)
Supplement: Supplementary file 1 — Supplementary information [file 41698_2026_1572_MOESM1_ESM.docx]

**Supplementary Material for *POLE* mutations in Colorectal Cancer Manuscript**

The Code to reproduce the analysis based on TCGA cohorts COAD and READ is publicly available: [*https://github.com/ngr-path/TCGA_POLE_CRC_Concurrent/blob/main/POLE_TCGA_CRC_Concurrent_Focus.R*](https://github.com/ngr-path/TCGA_POLE_CRC_Concurrent/blob/main/POLE_TCGA_CRC_Concurrent_Focus.R)

***Supplementary Data 1. Clinical and molecular characteristics of TCGA colorectal cancers with POLE alterations***

This dataset contains all TCGA-COAD and TCGA-READ cases harboring *POLE* alterations (n = 43), including *POLE* variants, tumor mutational burden, MSIsensor scores, clinicopathological information, and co-mutations detectable by the AmpliSeq for Illumina Focus Panel.

***Supplementary Table S1. Clinicopathological Features of POLE-mutant CRCs.***

******

| ***Supplementary Table S2. Non-Exonuclease Domain Mutations and Tumor Mutational Burden in TCGA Colorectal Cancer*** | |
| --- | --- |
| ***POLE_Protein*** | ***TMB_nonsynonymous*** |
| *L1235I R1371** | *320.77* |
| *E2137K* | *140.47* |
| *K1170Nfs*49* | *135.76* |
| *V2152M R924H K1383R A2030Pfs*18* | *84.3* |
| *R231H* | *81.77* |
| *L698Cfs*94* | *79.83* |
| *X1517_splice T1052M* | *77.83* |
| *A2030Pfs*18* | *65.57* |
| *P1547S* | *64.2* |
| *P1547S* | *64.2* |
| *Y1003C* | *64.1* |
| *T1313M* | *62.17* |
| *Y473C* | *47.3* |
| *V2152M* | *45.77* |
| *R759C* | *40.73* |
| *D1214A* | *39.1* |
| *D1214A* | *39.1* |
| *D2166N* | *33.63* |
| *R1160H* | *19.13* |
| *D2013N* | *4.3* |
| *G628R* | *4.13* |
| *K778del* | *3.43* |
| *A2040V* | *2.9* |
| *D1752N* | *2.2* |
| *Y1003C* | */* |
| *E719K* | */* |


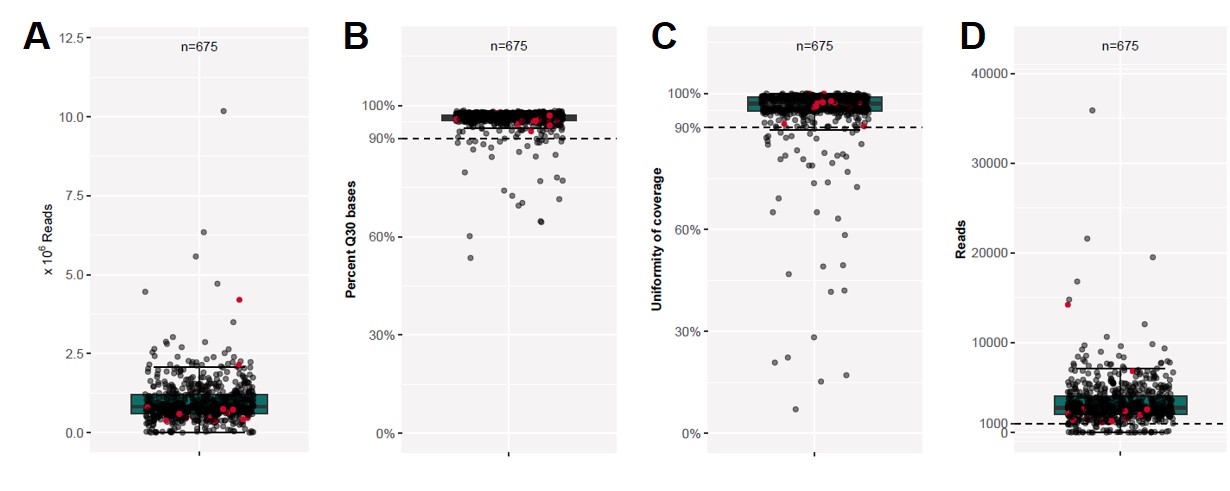


**Supplementary Fig. 1. QC metrics of all CRC cases in the UKA cohort. Boxplots displaying quality control metrics for all cases in the cohort.** *A)* Total passing filter reads with minimum threshold of 300.000 reads. *B)* Percentage of Q30 bases with a quality cutoff of 90%. *C)* Uniformity of coverage with a quality cutoff of 90%. *D)* Mean amplicon coverage with a minimum threshold of 1.000 reads. Cases suspected to be ultra-hypermutated due to pathogenic *POLE* mutations are highlighted in red.

CRC, colorectal cancer; QC, quality control; UKA, University Hospital Augsburg.


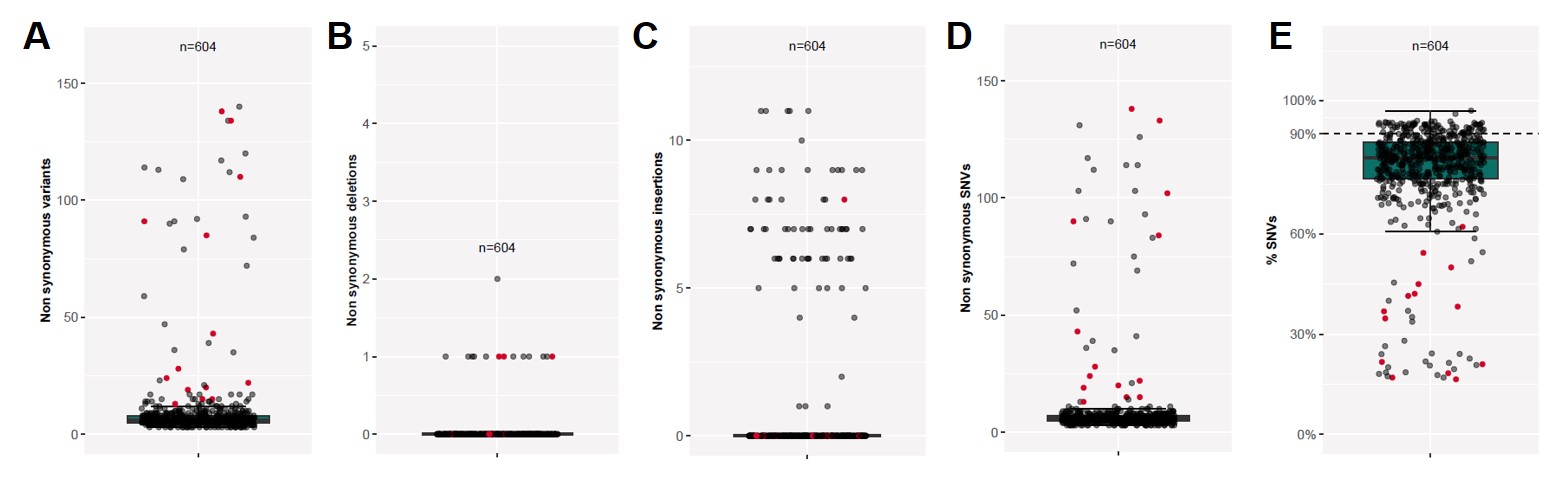


**Supplementary Fig. 2. Variant metrics contributing to the mutational load after quality control.** Boxplots displaying the distribution of variants contributing to the mutational burden across cases after quality control. *A)* Number of non-synonymous variants per case. *B)* Number of non-synonymous deletions per case. *C)* Number of non-synonymous insertions per case. *D)* Number of non-synonymous SNVs per case. *E)* Percentage of SNVs per case that are represented in dbSNP. Cases suspected to be ultra-hypermutated due to pathogenic *POLE* mutations are highlighted in red.

dbSNP, single nucleotide polymorphism database; POLE, DNA polymerase ε; SNVs, single nucleotide variants.

**
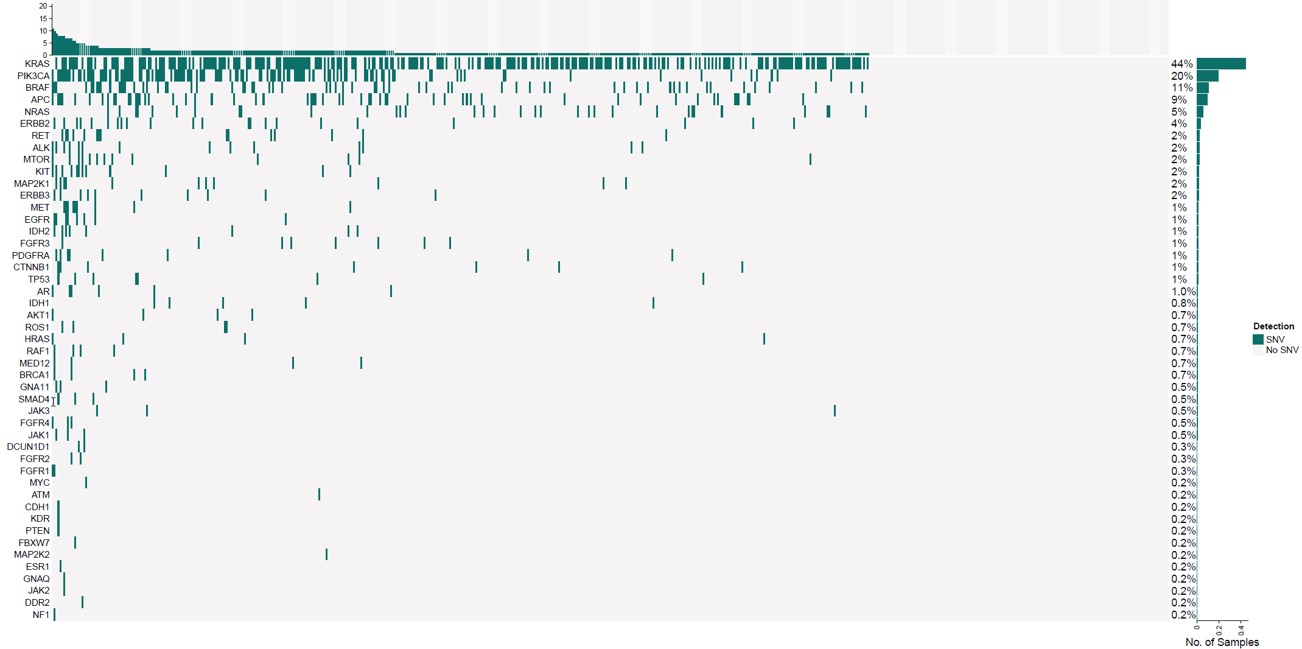
**

**Supplementary Fig. 3. Oncoprint of SNVs in the UKA CRC Cohort.** Oncoprint visualization of cases (n=604) with SNVs that passed quality control and were classified as pathogenic, likely pathogenic, or VUS. The left panel lists the genes included in the AmpliSeq Focus Panel for Illumina. The top panel displays the number of detected variants per case. The right panel shows the prevalence of variants per gene across the cohort, expressed as a percentage of cases affected. Dark green bars indicate the presence of SNVs in individual cases, while the absence of variants is shown in light gray.

SNVs, single nucleotid variants; UKA, University Hospital of Augsburg; VUS, variants of unknown significance

**Supplementary Fig. 4. Representative immunohistochemical stains for MMR proteins in of our *POLE-*mutant CRC.** All our *POLE*-mutant CRCs showed a retained expression of all four MMR markers *(A-D)*. and therefore, were classified as pMMR. Strong nuclear expression of cancer cells could be observed. Positive stromal cells serve as internal control.

CRC, colorectal cancer; POLE. DNA polymerase ε; pMMR, proficient mismatch repair.

**TCGA Colorectal Cancer Cohort: POLE-altered cases & ‚virtual‘ small panel approach**

**n=42**

**Supplementary Fig. 5: Oncoprint visualization of SNVs in TCGA-COAD and READ.** In **42 of 43** *POLE*-altered CRCs in TCGA, we could detect SNVs that could be potentially covered by the AmpliSeq Focus Panel. We here included all genes in the gene list of the described panel but *also the off target calls* we observed in our UKA cohort (in total n=64). SNVs included were missense, splice site, nonsense as well as multi-hit mutations.

COAD, colonic adenocarcinoma; CRC, colorectal cancer; POLE, DNA polymerase ε; READ, rectal adenocarcinoma; SNVs, single nucleotide variants; TCGA, The-Cancer-Genome-Atlas.

**TCGA Colorectal Cancer Cohort: POLE-altered cases & ‚virtual‘ small panel approach**

**n=41**

**Supplementary Fig. 6. Oncoprint visualization of SNVs in TCGA-COAD and READ.** In **41 of 43** *POLE*-altered CRCs in TCGA. we could detect SNVs in genes covered by the AmpliSeq Focus Panel. We here included all genes in the gene list. SNVs included were missense, splice site, nonsense as well as multi-hit mutations.

COAD, colonic adenocarcinoma; CRC, colorectal cancer; POLE, DNA polymerase ε; READ, rectal adenocarcinoma; SNVs, single nucleotide variants; TCGA, The-Cancer-Genome-Atlas.

**Supplementary Fig. 7. TCGA-CRC cases with POLE alteration and no concurrent gene alteration in genes covered by the AmpliSeq for Illumina Focus Panel.** In only two CRC cases within TCGA cohorts COAD and READ no concurrent alteration in a gene covered by the small NGS panel used in this study (Focus Panel) could be observed. Those cases showed both low TMB and only in one case partial mucinous differentiation *(A)*, with a predominant conventional adenocarcinoma NOS morphology in both cases *(A, B).*

COAD, colonic adenocarcinoma; CRC, colorectal cancer; POLE, DNA polymerase ε; TMB, tumor mutational burden; NGS, next generation sequencing; NOS, not otherwise specified.

Freqency in %

Freqency in %

**Supplement Fig. 8. The graphs represent the nucleotide substitution pattern observed in the TCGA cohort. Samples** included were *POLE*-altered cases with co-mutations in genes covered by the AmpliSeq Focus Panel gene list (n=41). Upper left panel shows nucleotide substitution pattern in general. Upper right panel shows relative distribution of transitions (Ti) and transversions (Tv) mutations. Lower panel shows nucleotide substitution pattern per case.

CRC, colorectal cancer; POLE, DNA polymerase ε; TCGA, The-Cancer-Genome-Atlas.

**Supplement Fig. 9. Heatmap representation of somatic interactions between mutated genes in TCGA-CRC cohort.** The co-occurrence and mutual exclusivity of mutations are visualized. with statistical significance indicated by asterisks (* P < 0.01. • P < 0.05). Colors represent

-log10(p-values). with green indicating co-occurrence and brown representing mutually exclusive mutations. Somatic interaction. *A)* Focusing on SNVs including all genes detected in our UKA CRC cohort by targeted panel sequencing (also off-target calls. n=64). *B)* Focusing on SNVs in genes covered by the AmpliSeq Focus Panel gene list. Only samples with *POLE* alterations and concurrent potentially detectable alterations were included.

CRC, colorectal cancer; POLE, DNA polymerase ε; SNVs, single nucleotide variants; TCGA, The-Cancer-Genome-Atlas.


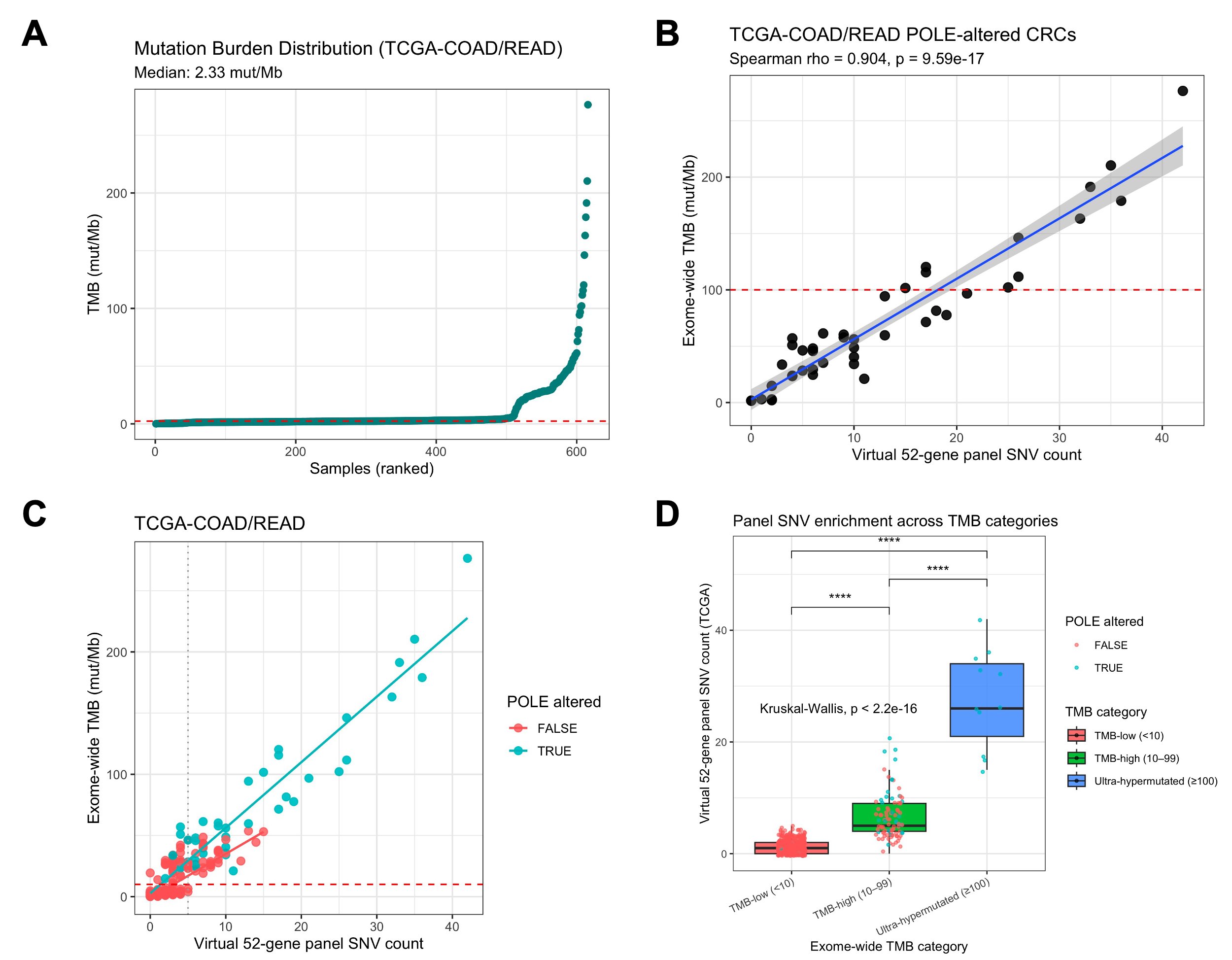


**Supplementary Figure 10. Relationship between virtual 52-gene panel mutation counts and exome-wide tumor mutational burden (TMB) in TCGA colorectal cancer.**

Exome-wide TMB was calculated from non-synonymous variants per megabase using *maftools*, and a virtual panel mutation count was derived by restricting mutations to genes included in the 52-gene panel.

*A)* Distribution of exome-wide TMB across TCGA-COAD/READ tumors ranked by mutation burden (median 2.33 mut/Mb). The dashed horizontal line indicates the commonly used clinical threshold for TMB-high (≥10 mut/Mb).

*B)* Correlation between the number of SNVs detectable within the virtual 52-gene panel and exome-wide TMB in *POLE*-altered colorectal cancers. Panel mutation counts strongly correlated with genome-wide TMB (Spearman ρ = 0.904, p<0.0001).

*C)* Correlation between virtual panel SNV count and exome-wide TMB across all TCGA colorectal cancers (Spearman ρ = 0.673, p<0.0001). *POLE*-altered tumors cluster at the upper end of both panel SNV burden and TMB.

*D)* Panel SNV counts stratified by TMB category (<10, 10-99, ≥100 mut/Mb). Panel mutation burden increased stepwise with increasing genome-wide TMB (Kruskal-Wallis p<0.0001), supporting the use of small-panel SNV enrichment as a surrogate indicator of hypermutation. Individual *POLE*-altered tumors are highlighted.
